# Tick-borne bacterial agents in *Hyalomma asiaticum* ticks from Xinjiang Uygur Autonomous Region, Northwest China

**DOI:** 10.1186/s13071-024-06256-y

**Published:** 2024-04-02

**Authors:** Bing Zhang, Niuniu Zhang, Tao Zheng, Miao Lu, Bierk Baoli, Runda Jie, Xiao Wang, Kun Li

**Affiliations:** 1 School of Basic Medical Sciences, Xinjiang Medical University, Institute of Medical Sciences of Xinjiang Medical University, Xinjiang Key Laboratory of Molecular Biology for Endemic Diseases, Urumqi, 830011 Xinjiang Uygur Autonomous Region China; 2 Xinjiang 474 Hospital, China RongTong Medical Healthcare Group CO.LTD, Urumqi, 830011 Xinjiang Uygur Autonomous Region China; 3 National Institute for Communicable Disease Control and Prevention, Chinese Center for Disease Control and Prevention, Changping District, Beijing, 102206 China; 4 Animal Disease Prevention and Control Center of Mulei Kazak Autonomous County, Changji Hui Autonomous Prefecture, Xinjiang Uygur Autonomous Region China

**Keywords:** *Hyalomma asiaticum*, *CandidatusBorrelia hyalommii*, *Rickettsia sibirica* subsp. mongolitimonae, *CandidatusAnaplasma camelii*, Xinjiang, Recombination

## Abstract

**Background:**

*Hyalomma* ticks are widely distributed in semi-arid zones in Northwest China. They have been reported to harbor a large number of zoonotic pathogens.

**Methods:**

In this study, a total of 334 *Hyalomma* asiaticum ticks infesting domestic animals were collected from four locations in Xinjiang, Northwest China, and the bacterial agents in them were investigated.

**Results:**

A putative novel Borrelia species was identified in ticks from all four locations, with an overall positive rate of 6.59%. *Rickettsia sibirica* subsp. mongolitimonae, a human pathogen frequently reported in Europe, was detected for the second time in China. Two *Ehrlichia* species (*Ehrlichia minasensis* and *Ehrlichia* sp.) were identified. Furthermore, two Anaplasma species were characterized in this study: *Candidatus* Anaplasma camelii and Anaplasma sp. closely related to *Candidatus* Anaplasma boleense. It is the first report of *Candidatus* Anaplasma camelii in China.

**Conclusions:**

Six bacterial agents were reported in this study, many of which are possible or validated pathogens for humans and animals. The presence of these bacterial agents may suggest a potential risk for One Health in this area.

**Graphical Abstract:**

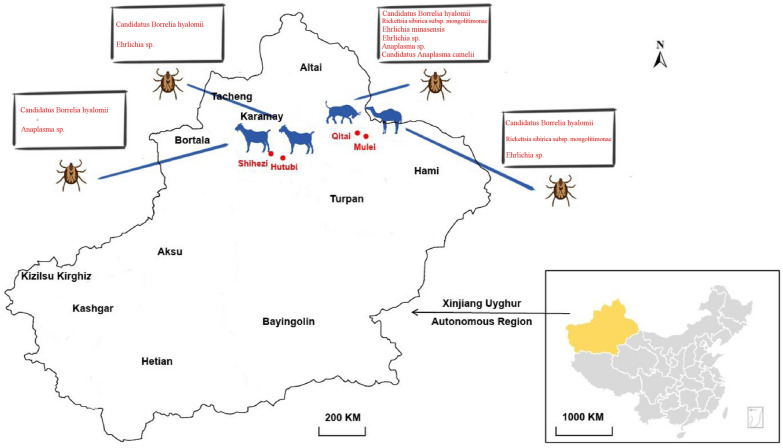

**Supplementary Information:**

The online version contains supplementary material available at 10.1186/s13071-024-06256-y.

## Background

In past decades, tick and tick-borne diseases have posed a great threat to livestock productivity and public health globally. To date, 20 tick genera belonging to three families (Ixodidae, Argasidae, and Nuttalliellidae) have been defined worldwide [1, 2]. Of those, the ixodid tick species of the genus *Hyalomma* is mainly distributed in the semi-arid zones (such as semidesert steppes, savannas, scrubland, etc.) of African, Asian, and Mediterranean countries [3]. A total of 27 *Hyalomma* species are recognized, presenting on all continents except in North and South America [3]. Most *Hyalomma* ticks are three-host ticks; in each of their three stages, they must find a new host to take the blood meal [4]. The hosts of *Hyalomma* ticks are mainly domestic or wild animals. Occasionally, humans can also become their accidental hosts.

*Hyalomma* ticks (*Hyalomma anatolicum*, *Hy. marginatum*, *Hy. rufipes*, *Hy. asiaticum*, etc.) have been recognized as the competent vector of Crimean Congo hemorrhagic fever virus (CCHFV), which causes an acute and often fatal hemorrhagic fever in humans, with the reported fatality rate ranging from 3 to 30% [5]. Apart from CCHFV, many viruses, bacteria, and protozoan agents have been reported in *Hyalomma* ticks, such as West Nile virus, Rift Valley fever virus, *Rickettsia rickettsii*, *R. aeschlimannii*, *R. sibirica*, *Anaplasma phagocytophilum*, *Ehrlichia canis*, *Coxiella burnetii*, *Borrelia turcica*, *Babesia occultans*, and *Theileria ovis* [4, 5], many of which cause zoonotic diseases. However, for many of these agents, the roles of *Hyalomma* ticks in their transmission and maintenance are still to be determined.

In China, the major species in the genus *Hyalomma* are *Hyalomma scupense*, *Hy. asiaticum*, *Hy. dromedarii*, *Hy. anatolicum*, and *Hy. marginatum* [6]. They are mainly distributed in northwestern provinces such as Xinjiang, Gansu, and Inner Mongolia. Although many investigations studying tick-borne agents in *Hyalomma* ticks have been carried out in China, comprehensive studies on bacterial agents are still limited given their vast geographical distribution. In Xinjiang Uygur Autonomous Region, with an area of 1,664,900 km^2^, although some studies reported the agents in *Hyalomma* ticks, most are still preliminary and lack further genetic characterization. In this study, we collected ticks in four locations in Xinjiang and investigated the tick-borne bacterial agents in them.

## Methods

### Sample collection and DNA extraction

**Fig. 1:**
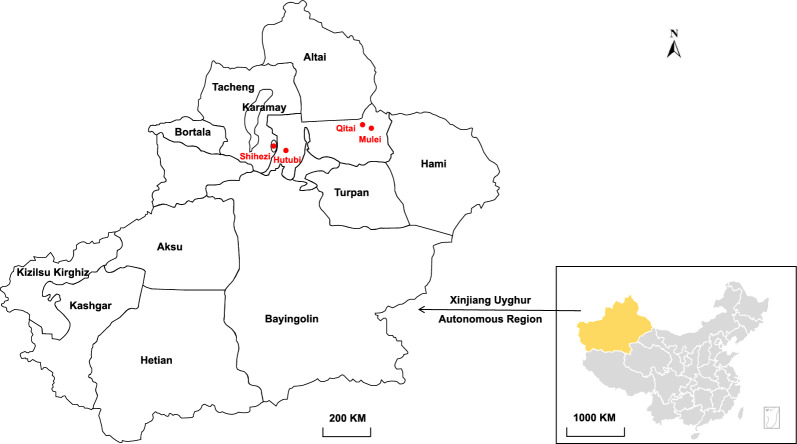
Map showing the four locations where the *Hyalomma asiaticum* ticks were collected in Xinjiang

From April to May 2023, ticks were collected from the body surface of domestic animals in four locations in Xinjiang Uygur Autonomous Region (Qitai, Mulei, and Hutubi counties of Changji Hui Autonomous Prefecture and 150 Regiment of Shihezi City) (Fig. 1). Ticks were carefully removed from domestic animals (camels, sheep, and cattle) using tweezers. All ticks were brought to the laboratory alive. Morphological identification was performed to determine their species initially [7]. After washing twice using phosphate buffer, all the ticks were ground into homogenate and subjected to DNA extraction using the TIANamp genomic DNA Extraction Kit (TIANGEN Co.). The tick species was confirmed by amplifying and sequencing the mitochondrial cytochrome C oxidase subunit I (*COI*) gene (primers shown in [8]).

### Detection of *Borrelia* and analysis

All the DNA samples were screened for *Borrelia* using primers amplifying the *flaB* (flagellin B) gene, with a PCR product of approximately 400 bp. For the positive samples, partial 16S rRNA (approximately 1200 bp) sequences were amplified for further characterization (primers shown in [9]). All obtained sequences were aligned with those in the GenBank Database by BLASTn to determine the nucleotide identity. For phylogenetic analysis, reference sequences were downloaded from the GenBank Database and manually aligned with those recovered in this study using the MegAlign program. The maximum likelihood (ML) tree was then constructed using PhyML v3.0 under the best-fit substitution model determined by MEGA 7.0 [10].

### Detection and analysis of *Rickettsia* and Anaplasmataceae bacteria

*Rickettsia* and Anaplasmataceae bacteria (mainly *Anaplasma* and *Ehrlichia*) were detected by hemi-nested or nested PCR targeting a partial region of the 16S rRNA gene (approximately 900 bp for *Rickettsia* and 500 bp for Anaplasmataceae bacteria) [11]. After sequencing and alignment by BLASTn, the bacterial species were initially determined. For further characterization, the *gltA* (citrate synthase) and *groEL* (60 kDa chaperonin) sequences were recovered from samples positive for *Rickettsia*, *Ehrlichia*, and *Anaplasma* using degenerate primers (primers shown in [11]) or specific primers (primers shown in [12]). The *ompA* sequences were additionally obtained from *Rickettsia* strains, while longer 16S sequences (approximately 800 bp) were obtained from *Anaplasma* strains [11]. All the sequences were genetically and phylogenetically analyzed as indicated above.

## Results

### Samples

In the summer of 2023, 334 ticks were collected in four locations in Xinjiang. In Mulei County, 72 ticks were collected from camels. In Hutubi County and Shihezi City, 46 and 96 ticks were collected from goats, respectively. In Qitai County, 120 ticks were collected from cattle. All ticks were initially identified to be *Hy. asiaticum* based on their morphology by using taxonomic keys. This result was confirmed by sequencing and analysis of the *COI* sequences (all sequences showed > 99% identities to *Hy. asiaticum*).

### *Borrelia* sp.

**Fig. 2:**
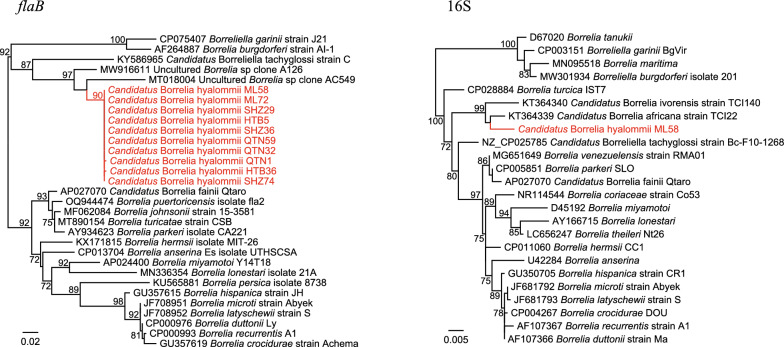
Phylogenetic trees based on the *flaB* and 16S sequences of *Candidatus Borrelia hyalommii* strains

*Borrelia* was detected in *Hy. asiaticum* ticks from all four locations, with a total positive rate of 6.59% (22/334). The positive rates in ticks from four locations varied from 5.56% to 7.50% (Table 1). Genetic analysis of the *flaB* gene indicated that they share 99.76–100% nucleotide identity from each other, indicating that all of them represent the same species. When compared with sequences in the GenBank Database, they have only highest 91.69–91.93% identities to uncultured *Borrelia* strains (clone AC549, AC444, AO17, AC425) detected in Brazil and 86.51–86.75% identities to *Borrelia johnsonii* strain 15-3581 (MF062084.1). However, only one 16S sequence was successfully recovered, which shows 98.82% identity to *Candidatus* *Borrelia africana* strain TCI22 (KT364339.1), 98.28% to *Candidatus* *Borrelia ivorensis* strain TCI140 (KT364340.1), and 97.81% to *Borrelia turcica* IST7 (CP028884.1). In the phylogenetic trees (Fig. 2), these strains form distinct clades, suggesting that they represent a putative novel species. Herein, we tentatively name it “*Candidatus* *Borrelia hyalommii*”.

**Table 1:** Prevalence of tick-borne bacterial agents in *Hyalomma asiaticum* in four locations of Xinjiang Uygur Autonomous Region

Bacterial species		Qitai	Mulei	Hutubi	Shihezi	Total
*Borrelia*	*Candidatus* Borrelia hyalomii	7.50% (9/120)	5.56% (4/72)	6.52% (3/46)	6.25% (6/96)	6.59% (22/334)
*Rickettsia*	*Rickettsia sibirica* subsp. mongolitimonae	0.83% (1/120)^a^	2.78% (2/72)	0.00% (0/46)	0.00% (0/96)	0.90% (3/334)
*Ehrlichia*	*Ehrlichia minasensis*	3.33% (4/120)	0.00% (0/72)	0.00% (0/46)	0.00% (0/96)	1.20% (4/334)
	*Ehrlichia* sp.	2.50% (3/120)	1.39% (1/72)	8.70% (4/46)	0.00% (0/96)	2.40% (8/334)
*Anaplasma*	*Anaplasma* sp.	7.50% (9/120)	0.00% (0/72)	0.00% (0/46)	4.17% (4/96)	3.89% (13/334)
	*Candidatus* Anaplasma camelii	3.33% (4/120)	0.00% (0/72)	0.00% (0/46)	0.00% (0/96)	1.20% (4/334)

^a^Positive samples/total samples

### *Rickettsia* sp.

**Fig. 3:**
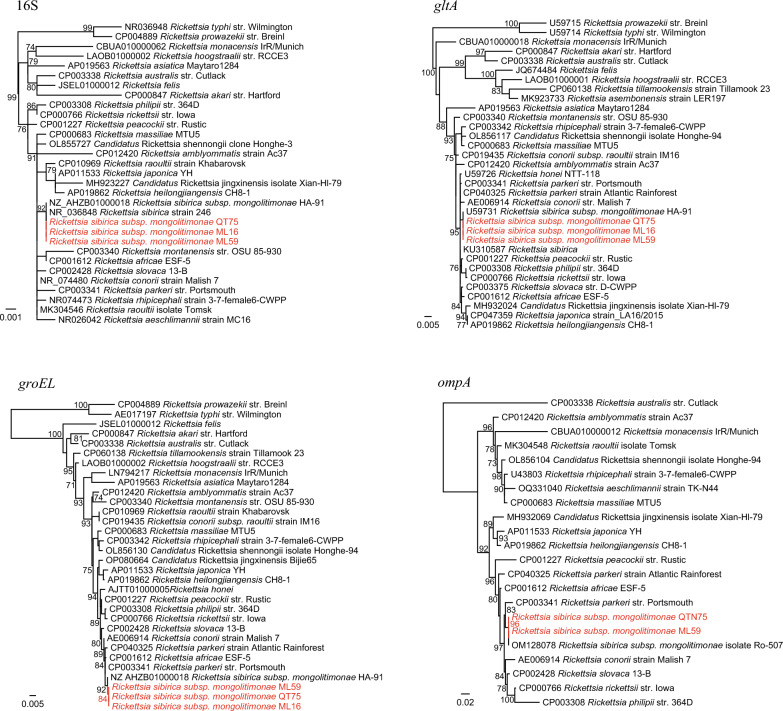
Phylogenetic trees based on the 16S, *gltA*, *groEL*, and *ompA* nucleotide sequences of *Rickettsia sibirica* subsp. mongolitimonae strains

Three ticks tested positive for *Rickettsia*, one from Qitai County and two from Mulei County. The 16S sequences are 100% identical to *R. sibirica* strain 246 and *R. sibirica* subsp. mongolitimonae HA-91. The *gltA*, *groEL*, and *ompA* sequences were also analyzed, which are closely related to *R. sibirica* subsp. mongolitimonae strains (Fig. 3) with 100%, 99.90%, and 100% nucleotide similarities. Therefore, these strains are determined to be *R. sibirica* subsp. mongolitimonae.

### *Ehrlichia* spp.

**Fig. 4:**
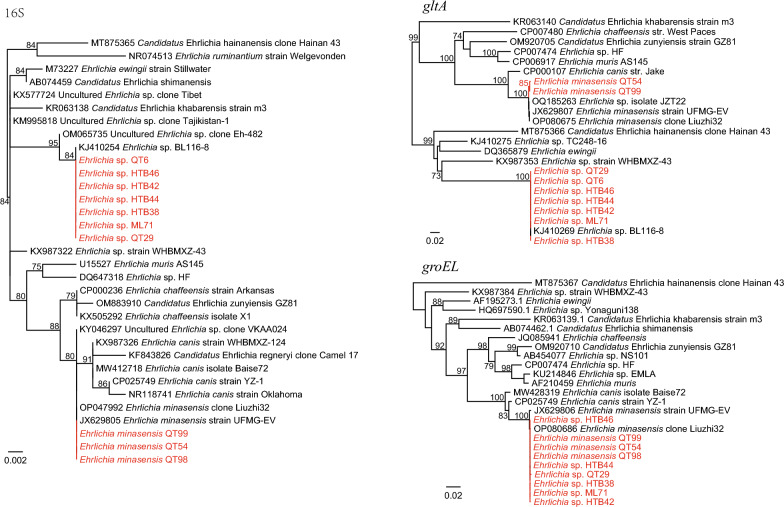
Phylogenetic trees based on the 16S, *gltA*, and *groEL* gene sequences of the *Ehrlichia* strains

A total of 12 *Ehrlichia* strains were detected in the 334 ticks (3.59%), representing two species. Of those, four strains (QT54, QT88, QT98, QT99) from Qitai County were determined to be *E. minasensis*, with their 16S, *gltA*, and *groEL* sequences showing 100%, 99.40–99.60%, and 100% identities to *E. minasensis* strains. Analysis of the other eight *Ehrlichia* strains indicated that they are closely related to *Ehrlichia* sp. BL116-8, which was also identified in ticks from Xinjiang. Their 16S and *gltA* sequences are all 100% identical to *Ehrlichia* sp. BL116-8. Interestingly, as shown in the phylogenetic tree, although their 16S and *gltA* are distinct from *E. minasensis*, all their *groEL* sequences are closely related to those of *E. minasensis* strains (99.81–100% nucleotide identity) (Fig. 4).

### *Anaplasma* spp.

**Fig. 5:**
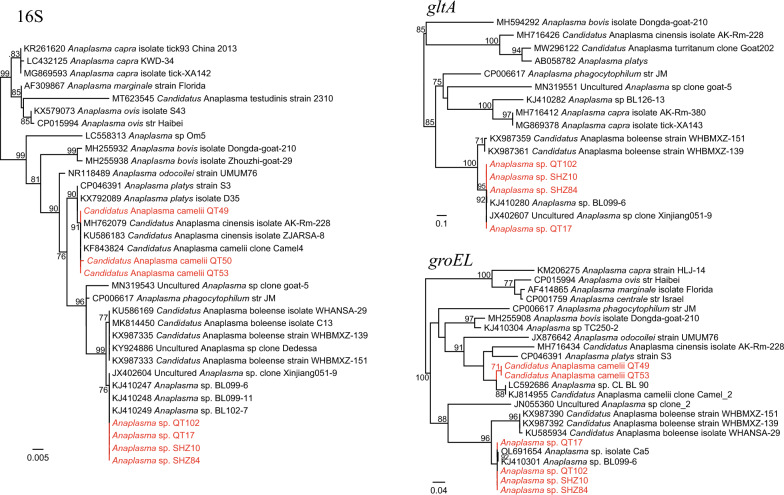
Phylogenetic trees based on the 16S, *gltA*, and *groEL* gene sequences of the *Anaplasma* strains

Based on the 16S, *gltA*, and *groEL* sequences, two *Anaplasma* species were initially determined: One is almost identical to *Anaplasma* sp. BL099-6 previously reported in Bole City of Xinjiang. Meanwhile, it is also closely related to *Candidatus* *Anaplasma boleense* in the phylogenetic trees. In our opinion, it might be reasonable to classify it as a subspecies or variant of *Candidatus* *Anaplasma boleense*. For the other species, only 16S and *groEL* sequences were successfully obtained. The 16S sequences are highly homologous to *Candidatus* *Anaplasma camelii* and *Candidatus* *Anaplasma cinensis* (both have 99.87–100% identity), while the *groEL* sequences have highest 95.44% nucleotide similarity to *Anaplasma* sp. CL BL 90 and 94.90% to *Candidatus* *Anaplasma camelii* strains. In the phylogenetic trees, they are also closely related to *Candidatus* *Anaplasma camelii* strains (Fig. 5). Based on these data, we propose that they should be a variant of *Candidatus* *Anaplasma camelii* despite the absence of *gltA* sequences.

## Discussion

*Hyalomma* ticks are important vectors of human pathogens in Northwest China. However, extensive and comprehensive studies on the bacterial pathogens they harbor are still lacking. In this study, we collected 334 *Hy. asiaticum* ticks from four locations in Xinjiang, and six bacterial agents were detected and identified.

Since 2014, the former genus *Borrelia* has been divided into two genera: the genus *Borrelia* containing the members of the relapsing fever *Borrelia* and the genus *Borreliella* containing the Lyme disease *Borreliella* [13]. In China, *Borreliella burgdorferi *sensu lato, the agent of Lyme disease, has been frequently detected in multiple tick species [6, 14]. However, reports on members of the current genus *Borrelia* have been quite few in Xinjiang. In 2020, Zhao et al. reported a previously undescribed *Borrelia* species in *Rhipicephalus turanicus* from Xinjiang based on the *groEL* sequence [15]. In 2022, a nationwide survey revealed the remarkable genetic diversity of relapsing fever *Borrelia* in ticks from China [16]. However, no *Hy. asiaticum* has been reported to harbor relapsing fever *Borrelia*. In this study, we detected an undescribed *Borrelia* species in *Hy. asiaticum* ticks from all four locations, suggesting its wide distribution in Xinjiang. Analysis of its 16S and *flaB* genes indicated that it is genetically distant from known *Borrelia* species. Its pathogenicity to humans is still unclear and warrants further investigation.

The only *Rickettsia* species identified in this study is *R. sibirica* subsp. mongolitimonae, a member of the spotted group *Rickettsia*. *Rickettsia sibirica* subsp. mongolitimonae has long been recognized as a human pathogen causing fever, lymphangitis, septic shock, encephalitis, and retinal vasculitis [17–19]. Although it was first isolated from *Hy. asiaticum* ticks in Inner Mongolia of China [20], it was mostly reported in European and African countries, such as France, Spain, Portugal, Greece, Algeria, South Africa, and Cameroon [17–19, 21, 22]. As previously reported, *R. sibirica* has been reported in Xinjiang as well as elsewhere in China; however, almost all of them are *R. sibirica* subsp. sibirica or only identified to species level [23–26]. So far as we know, this may be the second report of *R. sibirica* subsp. mongolitimonae in China.

Two *Ehrlichia* species were identified in this study. Intriguingly, the two *Ehrlichia* species share almost the same *groEL* gene sequences (99.81–100%) despite the obvious discrepancy in their 16S and *gltA* sequences. We suppose that this may result from frequent recombination between different *Ehrlichia* species, which may be the driving force of their adaptivity and virulence. Besides, so far as we know, this is the first description of *E. minasensis* in Xijiang. *Ehrlichia minasensis* is a tick-borne agent infecting cattle, cervids, and dogs [27]. Infection with *E. minasensis* in calf results in clinical manifestations such as fever, depression, lethargy, and thrombocytopenia [28]. It should be noted that Xinjiang is one of the most important provinces of animal husbandry in China, and the presence of *E. minasensis* may pose a great threat to animal health. Surveillance in domestic animals and vector control measures should be carried out to prevent its transmission. Furthermore, although the two *Ehrlichia* species have not been reported to infect humans, whether the recombination has any effects on their pathogenicity is interesting.

*Candidatus* *Anaplasma camelii* was first described in one-humped camels from Morocco in 2017 [29]. Since then, it has been detected in camels and their associated ectoparasites (camel keds and ticks) from Kenya and Saudi Arabia [30, 31]. In 2018, it was also detected in cattle from Peninsular Malaysia [32], suggesting its wide geographic distribution and wide host range. In this study, it is of interest that all *Candidatus* *Anaplasma camelii* strains were detected in *Hy. anatolicum* ticks infesting apparently healthy cattle from Qitai County. There may be two possibilities: (1) The *Hy. anatolicum* ticks may be vectors of *Candidatus* *Anaplasma camelii*. Previously, only one study reported the presence of *Candidatus* *Anaplasma camelii* in ticks. It has been demonstrated that camel keds (*Hippobosca camelina*) act as vectors of this *Anaplasma* species [33]. Instead, the role of *Hy. anatolicum* ticks in the transmission of *Candidatus* *Anaplasma camelii* has never been proved. (2) The detected DNA of *Candidatus* *Anaplasma camelii* is from the blood of cattle infected with it. Namely, cattle may be the reservoir of *Candidatus* *Anaplasma camelii*. This is consistent with a previous study in Peninsular Malaysia [32]. Both possibilities are worth further consideration and investigation. This is also the first report of *Candidatus* *Anaplasma camelii* in China. The presence of two *Anaplasma* species in this study should raise concern in Xinjiang due to their veterinary importance.

There are also some limitations in the current study. All the agents were detected in ticks collected from domestic animals. The possibility cannot be ruled out that the agents were actually from the blood meal of ticks. In other words, it is still not clear whether the *Hy. asiaticum* ticks are the effective vector of these bacteria. In future studies, investigations of these agents in host-seeking ticks at different developmental stages and domestic animals are still needed.

## Conclusions

Our results indicated the distribution and diversity of tick-borne agents in *Hy. anatolicum* ticks from Xinjiang, Northwest China. Among the six bacterial agents identified in this study, one represents a putative novel species (*Candidatus* *Borrelia hyalommii*), one is a human pathogen (*Rickettsia sibirica* subsp. mongolitimonae), and some of the others are animal pathogens. Possible recombination has been observed between *Ehrlichia* species, which may contribute to their pathogenicity. Furthermore, we also reported the presence of *Candidatus* *Anaplasma camelii* in China for the first time. All these data suggested the potential risk for human and animal health in this area.

### Supplementary Information

**Additional file 1: Table S1.** Accession numbers of the 16S, *gltA*, and *groEL* sequences of *Borrelia*, *Rickettsia*, *Ehrlichia*, and *Anaplasma* strains in this study in the GenBank Database.

## Data Availability

All sequence files are available from the NCBI database (Accession Numbers shown in Additional file 1: Table S1).
