# Supplementary material for: Tick-borne bacterial agents in Hyalomma asiaticum ticks from Xinjiang Uygur Autonomous Region, Northwest China
Source: Parasit Vectors. 2024 Apr 2;17:167. doi: 10.1186/s13071-024-06256-y (PMC10985858; doi:10.1186/s13071-024-06256-y)
Supplement: Supplementary file 1 — Additional file 1: Table S1. Accession numbers of the 16S, gltA, and groEL sequences of Borrelia, Rickettsia, Ehrlichia, and Anaplasma strains in this study in the GenBank Database. [file 13071_2024_6256_MOESM1_ESM.docx]

Table S1. Genbank numbers of *Rickettsia*, *Anaplasma*, *Ehrlichia*, and *Borrelia* sequences obtained in this study.

|  | **Gene** | **Genbank numbers** | **Bacterial strain** |
| --- | --- | --- | --- |
| 1 | 16S | OR827290 | Rickettsia_sibirica_ML16 |
| 2 | 16S | OR827291 | Rickettsia_sibirica_ML59 |
| 3 | 16S | OR827292 | Rickettsia_sibirica_QT75 |
| 4 | gltA | OR828579 | Rickettsia_sibirica_ML16 |
| 5 | gltA | OR828580 | Rickettsia_sibirica_ML59 |
| 6 | gltA | OR828581 | Rickettsia_sibirica_QT75 |
| 7 | groEL | OR828582 | Rickettsia_sibirica_ML16 |
| 8 | groEL | OR828583 | Rickettsia_sibirica_ML59 |
| 9 | groEL | OR828584 | Rickettsia_sibirica_QT75 |
| 10 | ompA | OR828585 | Rickettsia_sibirica_ML59 |
| 11 | ompA | OR828586 | Rickettsia_sibirica_QT75 |
| 12 | 16S | OR878665 | Candidatus_Borrelia_hyalomii_ML58 |
| 13 | flaB | OR881981 | Candidatus_Borrelia_hyalomii_HTB5 |
| 14 | flaB | OR881982 | Candidatus_Borrelia_hyalomii_HTB36 |
| 15 | flaB | OR881983 | Candidatus_Borrelia_hyalomii_SHZ29 |
| 16 | flaB | OR881984 | Candidatus_Borrelia_hyalomii_SHZ36 |
| 17 | flaB | OR881985 | Candidatus_Borrelia_hyalomii_SHZ74 |
| 18 | flaB | OR881986 | Candidatus_Borrelia_hyalomii_QT1 |
| 19 | flaB | OR881987 | Candidatus_Borrelia_hyalomii_QT32 |
| 20 | flaB | OR881988 | Candidatus_Borrelia_hyalomii_QT59 |
| 21 | flaB | OR881989 | Candidatus_Borrelia_hyalomii_ML71 |
| 22 | 16S | OR878669 | Anaplasma sp._SHZ10 |
| 23 | 16S | OR878670 | Anaplasma sp._SHZ84 |
| 24 | 16S | OR878671 | Anaplasma sp._QT17 |
| 25 | 16S | OR878672 | Anaplasma sp._QT102 |
| 26 | 16S | OR878673 | Candidatus Anaplasma camelii_QT49 |
| 27 | 16S | OR878674 | Candidatus Anaplasma camelii_QT50 |
| 28 | 16S | OR878675 | Candidatus Anaplasma camelii_QT53 |
| 29 | gltA | OR881975 | Anaplasma sp._SHZ10 |
| 30 | gltA | OR881976 | Anaplasma sp._SHZ84 |
| 31 | gltA | OR881977 | Anaplasma sp._QT17 |
| 32 | gltA | OR881978 | Anaplasma sp._QT102 |
| 33 | gltA | OR881989 | Ehrlichia_sp._ML71 |
| 34 | gltA | OR881990 | Ehrlichia_sp._HTB38 |
| 35 | gltA | OR881991 | Ehrlichia_sp._HTB42 |
| 36 | gltA | OR881992 | Ehrlichia_sp._HTB44 |
| 37 | gltA | OR881993 | Ehrlichia_sp._HTB46 |
| 38 | gltA | OR881994 | Ehrlichia_sp._QT6 |
| 39 | gltA | OR881995 | Ehrlichia_sp._QT29 |
| 40 | gltA | OR881996 | Ehrlichia_minasensis_QT54 |
| 41 | gltA | OR881997 | Ehrlichia_minasensis_QT99 |
| 42 | groEL | OR881998 | Ehrlichia_sp._ML71 |
| 43 | groEL | OR881999 | Ehrlichia_sp._HTB38 |
| 44 | groEL | OR882000 | Ehrlichia_sp._HTB42 |
| 45 | groEL | OR882001 | Ehrlichia_sp._HTB44 |
| 46 | groEL | OR882002 | Ehrlichia_sp._HTB46 |
| 47 | groEL | OR882003 | Ehrlichia_sp._QT29 |
| 48 | groEL | OR882004 | Ehrlichia_minasensis_QT54 |
| 49 | groEL | OR882005 | Ehrlichia_minasensis_QT98 |
| 50 | groEL | OR882006 | Ehrlichia_minasensis_QT99 |
| 51 | 16S | OR887205 | Ehrlichia_minasensis_QT54 |
| 52 | 16S | OR887206 | Ehrlichia_minasensis_QT98 |
| 53 | 16S | OR887207 | Ehrlichia_minasensis_QT99 |
| 54 | 16S | OR887208 | Ehrlichia_sp._HTB38 |
| 55 | 16S | OR887209 | Ehrlichia_sp._ML71 |
| 56 | 16S | OR887210 | Ehrlichia_sp._HTB42 |
| 57 | 16S | OR887211 | Ehrlichia_sp._HTB44 |
| 58 | 16S | OR887212 | Ehrlichia_sp._HTB46 |
| 59 | 16S | OR887213 | Ehrlichia_sp._QT6 |
| 60 | 16S | OR887214 | Ehrlichia_sp._QT29 |
| 61 | groEL | NA | Anaplasma_sp._QT17 |
| 62 | groEL | NA | Anaplasma_sp._QT102 |
| 63 | groEL | NA | Anaplasma_sp._SHZ10 |
| 64 | groEL | NA | Anaplasma_sp._SHZ84 |
| 65 | groEL | NA | Candidatus_Anaplasma_camelii_QT49 |
| 66 | groEL | NA | Candidatus_Anaplasma_camelii_QT53 |
